# PSMA PET/CT in Castration-Resistant Prostate Cancer: Myth or Reality?

**DOI:** 10.3390/jcm12227130

**Published:** 2023-11-16

**Authors:** Luca Urso, Luca Filippi, Angelo Castello, Maria Cristina Marzola, Mirco Bartolomei, Corrado Cittanti, Luigia Florimonte, Massimo Castellani, Paolo Zucali, Alessio Bruni, Roberto Sabbatini, Massimo Dominici, Stefano Panareo, Laura Evangelista

**Affiliations:** 1 Department of Nuclear Medicine—PET/CT Center, S. Maria della Misericordia Hospital, 45100 Rovigo, Italy; luca.urso@unife.it (L.U.); mariacristina.marzola@aulss5.veneto.it (M.C.M.); 2 Nuclear Medicine Unit, Department of Oncohaematology, Fondazione PTV, Policlinico Tor Vergata University Hospital, Viale Oxford 81, 00133 Rome, Italy; luca.filippi@ptvonline.it; 3 Nuclear Medicine Unit, Fondazione IRCCS Ca’ Granda, Ospedale Maggiore Policlinico, 20122 Milan, Italy; luigia.florimonte@policlinico.mi.it (L.F.); massimo.castellani@policlinico.mi.it (M.C.); 4 Nuclear Medicine Unit, Onco-Hematological Department, University Hospital of Ferrara, 44124 Ferrara, Italy; m.bartolomei@ospfe.it (M.B.); ctc@unife.it (C.C.); 5 Department of Translational Medicine, University of Ferrara, 44121 Ferrara, Italy; 6 Department of Biomedical Sciences, Humanitas University, Via Rita Levi Montalcini 4, 20072 Milan, Italy; paolo.zucali@hunimed.eu (P.Z.); laura.evangelista@hunimed.eu (L.E.); 7 Department of Oncology, IRCCS Humanitas Research Hospital, Via Manzoni 56, 20089 Milan, Italy; 8 Radiotherapy Unit, Department of Oncology and Hematology, University Hospital of Modena, 41124 Modena, Italy; bruni.alessio@aou.mo.it; 9 Oncology Unit, Department of Oncology and Hematology, University Hospital of Modena, Via del Pozzo 71, 41124 Modena, Italy; sabbatini@unimore.it (R.S.); dominici.massimo@aou.mo.it (M.D.); 10 Nuclear Medicine Unit, Department of Oncology and Hematology, University Hospital of Modena, Via del Pozzo 71, 41124 Modena, Italy; panareo.stefano@aou.mo.it; 11 Nuclear Medicine Unit, IRCCS Humanitas Research Hospital, Via Manzoni 56, 20089 Milan, Italy

**Keywords:** prostate cancer, PCa, castration-resistant prostate cancer, PSMA PET, FDG PET, pitfalls, UBU, second primary neoplasm

## Abstract

Background: prostate-specific membrane antigen (PSMA) ligand PET has been recently incorporated into international guidelines for several different indications in prostate cancer (PCa) patients. However, there are still some open questions regarding the role of PSMA ligand PET in castration-resistant prostate cancer (CRPC). The aim of this work is to assess the clinical value of PSMA ligand PET/CT in patients with CRPC. Results: PSMA ligand PET has demonstrated higher detection rates in comparison to conventional imaging and allows for a significant reduction in the number of M0 CRPC patients. However, its real impact on patients’ prognosis is still an open question. Moreover, in CRPC patients, PSMA ligand PET presents some sensitivity and specificity limitations. Due to its heterogeneity, CRPC may present a mosaic of neoplastic clones, some of which could be PSMA−/FDG+, or vice versa. Likewise, unspecific bone uptake (UBU) and second primary neoplasms (SNPs) overexpressing PSMA in the neoangiogenic vessels represent potential specificity issues. Integrated multi-tracer imaging (PSMA ligand and [^18^F]FDG PET) together with a multidisciplinary discussion could allow for reaching the most accurate evaluation of each patient from a precision medicine point of view.

## 1. Introduction

Castration-resistant prostate cancer (CRPC) is a widely recognized clinical condition characterized by rising prostate-specific antigen (PSA) despite castrate levels of serum testosterone [1,2]. CRPC represents an advanced heterogeneous disease setting still associated with a severe prognosis [3]. There are two distinct clinical subtypes of CRPC: non-metastatic CRPC (nmCRPC) and metastatic CRPC (mCRPC). The primary distinguishing factor is the presence of metastatic lesions in conventional imaging, such as computed tomography (CT) or bone scan [4,5]. Over the past decade, there have been significant advancements in the management of CRPC, both in the metastatic and non-metastatic settings. However, the determination of the most suitable treatment approach is primarily guided by the currently available imaging techniques [4].

In recent years, the introduction of next-generation imaging (NGI) into clinical practice has profoundly impacted the management of CRPC. Prostate-specific membrane antigen (PSMA) ligand positron emission tomography (PET)/computed tomography (CT) has demonstrated tangible results across various aspects of (PCa) and has been incorporated into international guidelines for several different indications [6]. One such indication is the evaluation of nmCRPC in conventional imaging. In this patient group, PSMA ligand PET/CT has proven to enhance the detection of PCa lesions, offering a more precise assessment of the extent of the disease and resulting in significant stage reclassification [7,8,9]. Furthermore, PSMA ligand PET/CT has shown superiority over conventional imaging in assessing mCRPC. In this subset of patients, PSMA ligand PET/CT outperforms bone scans in detecting skeletal metastases and surpasses radiologic imaging (CT and magnetic resonance imaging—MRI) in evaluating lymph node and visceral metastases [10,11].

However, besides the aforementioned insights, there are still some open questions regarding the role of PSMA ligand PET/CT in CRPC, to which we have yet to find a clear answer. The objective of this review is to assess the clinical value of PSMA ligand PET/CT in patients with CRPC. We aim to analyze the current indications and address the most crucial issues related to its clinical application.

## 2. Materials and Methods

Critical literature research was performed up to 31 August 2023 using the following electronic databases: PubMed, Scopus, and Web of Science. The articles considered were those regarding PSMA ligand PET imaging in CRPC. Only papers in the English language were assessed. The literature retrieved was carefully screened and evaluated by three authors (L.U., L.F., A.C.). We identified 4 topics regarding current challenges related to the use of PSMA PET/CT in CRPC: (a) switch from M0 to M1 disease; (b) unspecific bone uptake (UBU); (c) dual-tracer (PSMA/FDG) imaging; and (d) second primary neoplasms. Relevant articles retrieved were subdivided and discussed according to these 4 topics.

## 3. Results

### 3.1. From M0 to M1 Castration-Resistant Prostate Cancer: Bargain or Lost Opportunity?

nmCRPC is a condition distinguished by increasing values of prostate-specific antigen (PSA), castrate testosterone levels, and the absence of detectable metastases in cross-sectional imaging (abdomen–pelvis CT) and bone scans (BS) [1]. Until 2018, the primary approach to treating nmCRPC relied on maximal androgen blockade achieved by combining first-generation anti-androgen (bicalutamide) with androgen deprivation therapy. More recently, nmCRPC has been revolutionized by the implementation of androgen signaling inhibitors (ARSIs), which were proven to delay the development of metastases in patients with nmCRPC and a PSA doubling time (PSAdt) ≤ 10 months [12]. In particular, some phase 3 clinical trials (SPARTAN, PROSPER, ARAMIS) resulted in the approval of multiple ARSI compounds, including enzalutamide, apalutamide, and darolutamide, by regulatory authorities such as the Food and Drug Administration (FDA) and European Agency of Medicine (EMA) for the effective clinical management of nmCRPC [12,13]. Nevertheless, it is important to emphasize that, in all the above-mentioned clinical trials, the determination of nmCRPC status relied on BS and CT rather than on the advanced NGI techniques, such as PET/CT using molecular tracers [14,15]. NGI offers significantly enhanced sensitivity and specificity compared to conventional imaging, particularly when performed with PSMA ligands [16]. In this context, a few published papers specifically investigated the impact of PET/CT with PSMA ligands in nmCRPC patients (Table 1).

In a multicenter retrospective analysis carried out by Fendler et al. [9], the impact of PSMA ligands was assessed in a large cohort (*n* = 200) of patients defined as nmCRPC according to conventional imaging, but deemed at high risk of developing metastases due to PSAdt (i.e., ≤10 months) or Gleason score at diagnosis (≥8). Notably, PSMA ligand PET resulted in positive findings in almost all cases (196/200, 98%), of whom 55% had distant metastases to extra-pelvic lymph nodes and bones (M1). Notably, among the metastatic patients, 29 subjects (15%) had a single lesion while 28 cases (14%) presented an oligometastatic condition. Furthermore, information on clinical management after PSMA-PET was available in 148 cases, 122 of whom received new treatments after the execution of the PET/CT scan. Most interestingly, the authors identified some clinical variables (such as PSA ≥ 5.5 ng/mL, pN1) predicting M1 status on PSMA-PET and employed the aforementioned M1 predictors in a post hoc analysis of a subgroup of patients with similar characteristics to those included in the SPARTAN clinical trial (SPARTAN-like subgroup). The authors found that apalutamide maintained its benefit for metastasis-free survival (MFS) in all patients, including the SPARTAN-like population.

Fourquet and coworkers [7] reported at least one PSMA-avid lesion in 90% of patients otherwise classified as nmCRPC at conventional imaging, and defined the presence of oligometastatic disease in 20% of cases. The authors reported a change in clinical management in many cases being considered appropriate in 78% of patients.

Weber and colleagues [18] focused their retrospective analysis on patients affected by “early” CRPC, defined as a condition characterized by a PSA level of less than 3 ng/mL. PSMA ligand PET resulted in a positive finding in 75% of patients, of whom 45% had M1 status, while CT alone detected prostate cancer lesions in 18 out of 55 (33%) patients. Notably, PSMA ligand PET was capable of identifying oligometastatic disease in 68% of cases; however, the impact on clinical management in the included patients was not reported.

Wang and colleagues [17] utilized a dual-tracer PET/CT approach, employing [^68^Ga]Ga-PSMA-11 and [^18^F]FDG to investigate the metabolic heterogeneity of the disease (i.e., PSMA−/FDG+) in early-stage progressive cases with PSA levels ≤ 2 ng/mL. In their prospective trial, the authors enrolled 37 patients with high-risk (PSAdt ≤ 10 months) early progressive nmCRPC. Overall, 114 lesions were detected among 29 out of 37 patients, indicating a notably high prevalence (73%) of N+/M+ disease; 81 exhibited PSMA+/FDG± uptakes, while the remaining 33 were PSMA−/FDG+.

Upon reviewing the existing literature on the role of PSMA ligand PET in nmCRPC, several key observations emerge. Firstly, it becomes apparent that PSMA ligand PET exhibits superior sensitivity in detecting positive lesions compared to conventional imaging methods such as BS and CT [10,11]. Furthermore, it can successfully identify patients with extra-pelvic metastases that might otherwise go undetected. Based on the findings reported in published papers, it appears that nmCRPC may be a rarity, and a substantial number of patients experience the ‘Will Rogers phenomenon’, a phenomenon characterized by stage migration attributed to advancements in technology or changes in staging algorithms [19]. Secondly, PSMA ligand PET can identify oligometastatic disease in a substantial number of patients, thereby laying the foundation for PET-guided stereotactic treatments [20]. Furthermore, PSMA ligand PET can prompt changes in clinical management, potentially shifting patients from radiotherapy to systemic treatments, and vice versa, depending on their specific needs [21]. While PSMA ligand PET is promising in the context of nmCRPC, it is essential to underscore that the existing studies are predominantly retrospective and have not thoroughly examined whether PSMA ligand PET, apart from detecting more lesions and influencing clinical management, confers any survival benefits, both in terms of progression-free and/or overall survival. Hence, within this perspective, this pioneering imaging modality still represents an untapped opportunity, and additional research—preferably prospective and with larger cohorts—is imperative to better evaluate the significance of PSMA-PET in nmCRPC. To date, no specific recommendations are available in this setting of disease, although a balance should be found among early detection, treatment approach, and outcome.

### 3.2. Unspecific Bone Uptake: Mind the Gap!

PSMA ligand PET imaging has gained a primary role both in primary staging and restaging of PCa patients [22,23]. Despite several PSMA molecules having been investigated, in recent years, those labeled with [^18^F] are progressively replacing [^68^Ga]-labeled compounds due to several advantages, including lower positron energy, better spatial resolution, and the lack of need for a generator [24]. In parallel, several pitfalls have been described in the literature following the introduction of PSMA agents. In particular, unspecific bone uptake (UBU) on [^18^F]F-PSMA-1007 PET has been reported in a considerable fraction of PCa patients, leading to a potential increase in false-positive metastases and, consequently, to inadequate treatments [25,26].

Arnfield et al. [27] investigated whether patients with UBU at [^18^F]F-PSMA-1007 represent a higher-risk category of PCa. Almost half of the patients (94/214) showed at least one UBU, although none of them met the criteria for malignant lesions after a median follow-up of 16 months. Moreover, they showed that an SUVmax cut-off value ≥ 7.2, achieved a sensitivity of 100%, and a specificity of 98.6% for bone metastases. Likewise, Grünig et al. [28] analyze the frequency, anatomical distribution, characteristics, and potential impact on treatment selection of UBU in 348 PCa patients undergoing [^18^F]F-PSMA-1007. Again, approximately 50% of patients showed UBU, with higher frequency when using digital PET/CT than analog scanners.

Few studies have evaluated the rates of false-positive findings, including UBU, between [^68^Ga]Ga-PSMA-11 and [^18^F]F-PSMA-1007 [24,29,30]. Initially, Rauscher et al. [24] retrospectively investigated 102 patients with biochemical recurrent PCa. Overall, [^18^F]-F-PSMA-1007 revealed five times more benign lesions than [^68^Ga]Ga-PSMA-11 (245 vs. 52, respectively), as well as SUVmax was significantly higher for [^18^F]F-PSMA-1007 than [^68^Ga]Ga-PSMA-11. Although the frequency of bone lesions was slightly higher for [^68^Ga]Ga-PSMA-11 (24% vs. 27%), in absolute terms, UBU was substantially higher on [^18^F]F-PSMA-1007 (36 vs. 6), predominantly in the ribs. Hoberuck et al. [29] compared [^68^Ga]Ga-PSMA-11 and [^18^F]F-PSMA-1007 intra-individually for unspecific lesions in 46 PCa patients. No significant difference between [^18^F]F-PSMA-1007 and [^68^Ga]Ga-PSMA-11 was found in the SUVmax of primary lesions, lymph nodes, and skeletal metastases. However, unspecific uptake in the lymph nodes, bones, and ganglia was significantly higher in patients who underwent [^18^F]F-PSMA-1007. Recently, Seifert et al. [30] investigated the frequency of UBU (defined as focal bone uptake with SUVmax > 4 and PSA < 5 ng/mL) and skeletal metastases in patients who had a [^18^F]F-PSMA-1007 (*n* = 409) and [^68^Ga]Ga-PSMA-11 (*n* = 383) for biochemical recurrence of PCa. Of note, [^18^F]F-PSMA-1007 showed a higher rate of UBU than [^68^Ga]Ga-PSMA-11 (140 vs. 64; *p* = 0.001), whereas the rate of bone metastases was not different between the two radiopharmaceuticals. Among patients with UBU on [^18^F]F-PSMA-1007, 17 also had [^68^Ga]Ga-PSMA-11 PET/CT, and 12 had an additional bone scintigraphy and whole-body MRI. UBU was considered a false-positive when seen only on [^18^F]F-PSMA-1007.

Despite the different reasons that have been raised to explain UBU, such as unconjugated fluorine or activated bone marrow immune cells, the etiology is still unknown. In a recent paper, Ninatti et al. [31] explored the potential association between osteoporosis and UBU at [^18^F]F-PSMA-1007. Body mass index (BMI) and bone density were lower in the patients with UBU, although not statistically significant. However, UBU has also been reported in other [^18^F]-PSMA-targeting tracers [32,33].

To summarize, the high incidence of UBU using [^18^F]F-PSMA-1007 compared to [^68^Ga]Ga-PSMA-11 is challenging. Therefore, the evaluation of the images should be carefully considered, also by analyzing the corresponding CT findings. Furthermore, a complementary evaluation with other available radiotracers, including historical PCa radiotracers (i.e., [^18^F]F-choline and [^18^F]F-Fluciclovine) may be helpful in challenging cases [34,35,36]. Hopefully, the availability of new radiotracers currently under experimental examination, such as [^18^F]F-FDHT, may also contribute as an additional resource available for the future [37]. To overcome the limitation of UBU when PSMA-based PET images are interpreted, we suggest considering the clinical history of the patients, previous traumatic accidents, as well as CT characteristics.

**Figure 1:**
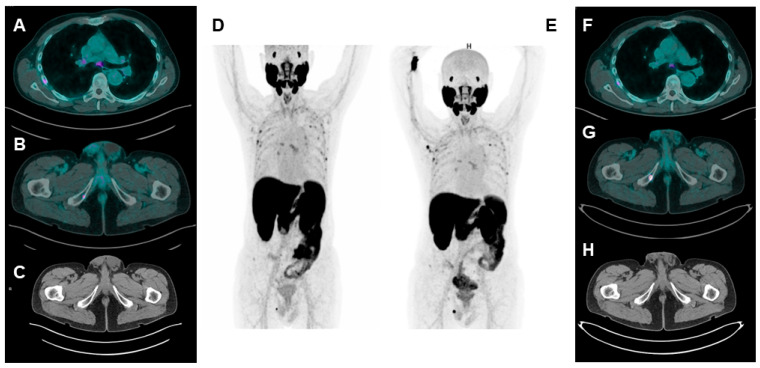
A patient with biochemical recurrence of PCa undergoing [^18^F]F-PSMA-1007 PET/CT (PSA = 0.22 ng/mL). Axial fused (**A**,**B**), CT axial (**C**), and maximum intensity projection (MIP) (**D**) images show multiple spots of focal uptake involving several ribs and the right ischio-pubic branch. The low PSA value leads to suspicion of UBU; therefore, a second [^18^F]F-PSMA-1007 PET/CT is performed 6 months later ((**E**): MIP image; (**F**,**G**): axial fused images; (**H**): CT axial image). While the rib uptakes appear stable for number, size and uptake intensity are suggestive of UBU; the uptake at the right ischio-pubic branch is considerably increased and an osteo-structural alteration can be detected at CT images. Combining imaging suspicion with increased PSA value (1.20 ng/mL), the lesion at the right ischio-pubic branch is considered a PCa metastasis.

Table 2 shows some hints for correct discrimination between UBU and bone metastases, and Figure 1 represents a case of mCRPC showing UBU at [^18^F]F-PSMA-1007.

### 3.3. Dual-Tracer PSMA/^18^F-FDG: Is It a Must?

**Figure 2:**
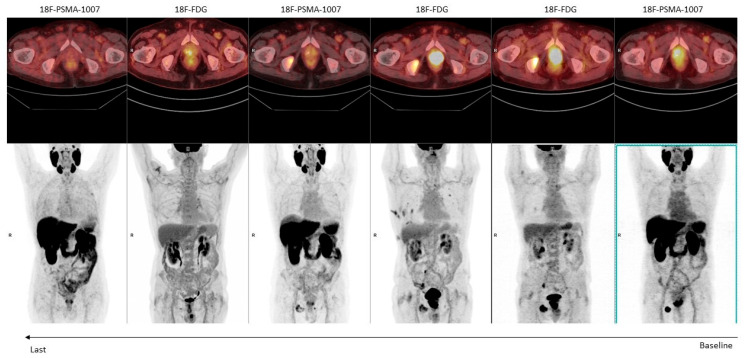
An 82-year-old man with a high-risk prostate cancer (Gleason score: 4 + 5) undergoing hormonal therapy. Serial [^18^F]FDG and [^18^F]-PSMA-1007 PET/CT examinations were performed to monitor the response to treatment (from right to left).

When considering CRPC, we are dealing with a very complex and heterogeneous disease [38]. The progressive development of castration resistance is defined by the acquisition of biochemical and genetic alterations that converge toward the selection of clones resistant to androgen deprivation therapy (ADT) [39]. Among these acquired alterations, we surely find the suppression of androgen receptor (AR) expression and activity. The final stage of CRPC is often represented by neuroendocrine dedifferentiation, which is an under-recognized, late, and aggressive manifestation of PCa (particularly associated with a high Gleason score), with a poor survival expectancy [40,41]. Available literature data have already hinted that neuroendocrine dedifferentiation is associated with a reduced PSMA expression and with the parallel activation of genes related to glucose uptake [40,42,43]. Therefore, these patients are characterized by a mosaic of lesions, potentially including PSMA-negative (PSMA−) and [^18^F]FDG-positive (FDG+) ones in molecular imaging (Figure 2). Notably, the identification of FDG+ lesions is a negative prognostic factor in mCRPC [44]. Bauckneht et al. [45] reported that the activation of [^18^F]FDG-related genes is usually parallel to a reduced expression of the FOLH1 gene, which encodes for PSMA expression. Interestingly, this genetic pattern was found to be also activated in some patients without neuroendocrine dedifferentiation [46]. These findings are consistent with several literature evidence reporting mCRPC patients without neuroendocrine dedifferentiation showing PSMA− lesions [42,46,47]. The main studies are reported in Table 3.

Chen et al. [42] compared the detection rate of [^68^Ga]Ga-PSMA-11 and [^18^F]FDG PET/CT in 56 mCRPC patients. [^68^Ga]Ga-PSMA-11 showed a higher detection rate than [^18^F]FDG (75% vs. 51.8%; *p* = 0.004), although 23.2% of patients showed at least 1 mismatched PSMA−/FDG+ lesion. In the study by Güzel et al. [49], the authors recommend dual-tracer PET imaging in mCRPC undergoing taxane chemotherapy, as 78.9% of visceral metastases were PSMA−/FDG+, representing a strong negative prognostic factor in multivariate Cox regression analysis. Similarly, semiquantitative parameters, such as the sum of total lesion glycolysis (TLG) and total lesion PSMA (TLP), were also predictors of shorter OS at multivariate analysis. Interestingly, in this study, patients were reported according to the Pro-PET scoring system, a six-tier integrated dual-tracer (PSMA ligands and [^18^F]FDG) PET/CT image scoring system ideated for mCRPC patients [50]. In the future, the validation of this scoring system could represent a valuable tool for reporting the imaging patterns of mCRPC patients by using a dual-tracer approach.

The majority of the remaining available data about double-tracer PET imaging in mCRPC patients is derivable from studies assessing patients’ eligibility for radioligand therapy (RLT) with [^177^Lu]Lu-PSMA. In the Lu-PSMA trial, 16% of patients were excluded from RLT due to mismatched PSMA−/FDG+ lesions [51]. Other studies are consistent with this finding, reporting that 18–33% of mCRPC patients show mismatched lesions in dual-tracer PET imaging [30,48]. Remarkably, FDG+ tumor volume is reported among prognostic factors in mCRPC patients receiving [^177^Lu]-PSMA [52].

Finally, in an ongoing prospective trial, Pouliot and colleagues [53] will assess the metabolic heterogeneity of 100 mCRPC patients with a triple-tracer PET imaging ([^68^Ga]Ga-PSMA-617, [^68^Ga]Ga-DOTATATE, and [^18^F]FDG). Hopefully, their results will clarify the different patterns of mCRPC patients from NGI.

Considering all these premises, which is the best NGI for mCRPC patients? Unfortunately, we actually do not have a clear answer to this crucial question. Nevertheless, current evidence pushes towards the possible synergic role of the dual-tracer PET imaging (PSMA ligands and [^18^F]FDG) in mCRPC. PSMA ligand PET/CT seems to have better diagnostic accuracy than [^18^F]FDG PET/CT, although it may not reflect patients’ whole burden of disease [42]. Nevertheless, the identification of PSMA−/FDG+ lesions is a negative prognostic factor that should be considered in particular for selecting patients as candidates for RLT with radiolabeled PSMA ligands [30,48,54]. According to Chen et al. [42], dual-tracer PET imaging could be suggested in mCRPC patients with high Gleason scores (≥8) and prostate-specific antigen (PSA) serum levels (i.e., >7.9 ng/mL in their cohort) in order to avoid undetected PSMA−/FDG+ lesions. In this subgroup of patients, the double-tracer PET imaging could increase the overall detection rate from 69.2% to 100%. However, this would considerably increase the number of PET/CT scan requests to nuclear medicine units, which would need a re-organization in terms of personnel and time resources. Moreover, the lack of cost-effectiveness analysis in the literature regarding dual-tracer imaging in mCRPC does not allow for finalizing conclusions on its applicability in daily clinical practice [55]. Therefore, the scarce evidence about this dual-imaging modality does not allow us to recommend the routine use of it in selecting patients who are candidates for [^177^Lu]Lu-PSMA.

### 3.4. Second Primary Neoplasms: Is PSMA Really “Prostate Specific”?

Despite its misleading name, PSMA is surely not a prostate-specific membrane antigen. The evidence that PSMA is overexpressed in the endothelium surface of the neoangiogenic vessels in several solid tumors has been largely demonstrated since the late 90 s [56]. In the most recent years, we saw an increased interest in the potential application of PSMA ligand imaging in non-prostate neoplasms, with encouraging results in renal cell carcinoma (RCC) and gliomas, while less favorable evidence for thyroid and gastro-enteric neoplasms exists [57,58,59,60,61,62,63,64]. Moreover, case reports or small-sized cohort studies have been published regarding PSMA-avid lesions from other neoplasms, including breast cancer, hepatocellular carcinoma, lung cancer, urothelial carcinoma, and salivary gland cancer [65,66,67,68,69].

Overall, the evidence that PSMA ligands are useful for imaging and—in a potential theranostic approach—treating other neoplasms is good news. Nevertheless, the finding that the “magic bullet” for PCa lacks specificity is also somewhat alarming. PCa is usually a neoplasm of the elder age. Moreover, CRPC is the last phase of the natural history of PCa, often occurring in multi-treated patients, several years after the diagnosis [70,71]. Considering all these risk factors, mCRPC patients have an increased risk of developing second primary neoplasms (SPNs), with a reported incidence rate ranging between 59 and 115 cases/year [72,73]. In a large cohort of 76,614 PCa patients, Chattopadhyay and colleagues [74] reported that 11.3% of patients received a diagnosis of SPN. In a cohort of 2234 CRPC patients, Saltus et al. [75] reported 172 cases of SPN, with an incidence rate of 5.9 cases per 100 persons/year. The most frequent SPNs were lung/bronchus cancer, followed by bladder and colorectal cancers (16.9%, 12.8%, and 12.2% of all SPNs, respectively). In another study by Mehtälä et al. [73], 100 SPNs were diagnosed among 693 mCRPC patients. Once again, the SPNs most frequently associated with PCa were bladder cancer, colorectal cancer, and lung cancer. On the other hand, in 3795 RCC patients with a diagnosis of SPN, Chakraborty et al. [76] reported that PCa was the most frequently associated malignancy, particularly in the first 6 months following the diagnosis of the renal disease.

**Figure 3:**
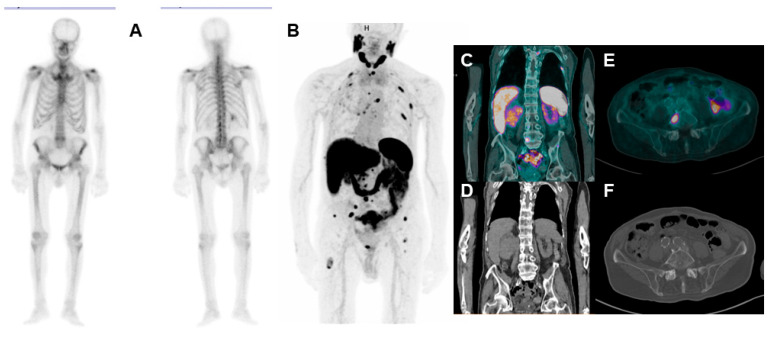
86-year-old CRPC patient undergoing bone scan ((**A**): anterior and posterior planar bone scan images) for rising PSA values (3.22 ng/mL). The scan shows no pathological uptakes; therefore, the patient underwent [^68^Ga]Ga-PSMA-11 PET/CT ((**B**): maximum intensity projection (MIP); (**C**,**D**): coronal-fused and CT images. (**E**,**F**): Axial-fused and CT images), with evidence of several bone focal uptakes, suggestive for metastases. Moreover, the scan shows a large area of increased uptake in the lower pole of the right kidney (SUVmax 6.8), associated with loco-regional lymph nodes with pathological uptake, compatible with renal SPN. In this case, a biopsy of bone lesions will be needed to discriminate their nature (PCa vs. RCC metastases).

Considering that most of these SPNs may overexpress PSMA in their neoangiogenic vessels, it can be expected to find patients with two different PSMA-avid primary malignancies in daily clinical practice (Figure 3). Indeed, several case reports have been published in the literature regarding patients with PCa and another PSMA-avid SPN [77,78,79,80,81,82]. This condition could represent a potential specificity issue in the Mcrpc setting, which is the PCa scenario most frequently associated with SPN. In particular, the identification of the primary tumor of a given PSMA-avid metastasis could represent a challenge for the nuclear medicine physician. Moreover, some PSMA-avid lesions could be in the differential diagnosis between PCa metastases and SPN (i.e., a lung nodule).

The nuclear medicine physician may usually have a hard time finding the right answer, and clinicians could be forced to perform a biopsy on uncertain lesions. SPN usually presents lower PSMA-avidity if compared to PCa; therefore, commonly used quantitative parameters (i.e., SUVmax) could allow for speculation in case of the impossibility of performing a biopsy [81]. However, RCC metastases have been documented with very high PSMA ligand uptake, comparable to those of PCa metastases [60,83,84]. A possible solution could be to perform [^18^F]FDG PET/CT and to make a double molecular imaging assessment of the patient’s pool of lesions. Nevertheless, in case of suspicious SPN, the results of PSMA PET/CT should be carefully considered in metastatic PCa patients, and a multidisciplinary board discussion should always be considered.

## 4. Conclusions

The development of a range of PSMA ligand PET in the diagnostic management of PCa patients surely represents one of the most relevant recent revolutions in the field of diagnostic imaging. However, some challenges still need to be solved, particularly in the CRPC setting. While PSMA ligand PET has demonstrated a higher detection rate in comparison to conventional imaging, allowing a significant reduction in the number of nmCRPC patients, its real impact on patients’ prognosis is still an open question [9,10,11]. Indeed, the upstaging from nm to mCRPC precludes some therapeutic possibilities, as conventional imaging was performed in the registering trials. Hopefully, new prospective trials will include PSMA ligand PET imaging in their workflows.

Moreover, in the CRPC setting, PSMA ligand PET presents some limitations in sensitivity and specificity. Due to its heterogeneity, CRPC may present a mosaic of neoplastic clones, some of which could be PSMA−/FDG+. Therefore, to have a reliable assessment of the whole burden of disease, dual-tracer imaging could be considered in mCRPC, particularly in those already subjected to multiple lines of treatment, or if neuroendocrine dedifferentiation is suspected [42,49].

Finally, UBU (at [^18^F]F-PSMA PET) and SPN represent specific issues for PSMA ligand PET in PCa patients. Their misinterpretation could be particularly relevant in mCRPC, determining a wrong treatment selection both in terms of timing and type of therapy. Once again, an integrated multi-tracer or multi-imaging approach could hypothetically provide the answer to this issue, although a cost-effective analysis is needed to reach a final recommendation. However, CRPC is a very complex and heterogeneous disease, and a multidisciplinary discussion should be encouraged to reach the most accurate evaluation of each patient from a precision medicine point of view.

## Figures and Tables

**Table 1:** Main findings of the selected papers on the applications of PSMA ligand PET/CT in nmCRPC.

Reference	Location/Year/	Study	N. of Patients	Primary Endpoint	PSMA Ligands	Comment
Fendler et al. [9]	Germany/USA 2019	Retrospective, investigator-initiated, multicenter	200	Detection rate of lesions, on a per-patient basis, by PSMA-PET.	^68^Ga-PSMA-11/ ^18^F-DCFPyL	Almost all cases showed positive findings in spite of negative conventional imaging: 55% of patients had distant metastases (M1).
Fourquet et al. [7]	France/2020	Retrospective	31	Impact of PSMA PET in the restaging of nmCRPC patients.	^68^Ga-PSMA-11	PSMA-PET detected at least 1 focus of tracer uptake in 90% of cases and changed clinical management in 87% of cases.
Wang et al. [17]	China/2021	Prospective, observational	37	To assess metabolic heterogeneity (PSMA+/FDG− disease) in early progressive nmCRPC.	^68^Ga-PSMA-11/ [^18^F]FDG	A total of 114 lesions were detected among 29 out of 37 nmCRPC patients. N+/M+ disease was detected in 73% of patients.
Weber et al. [18]	Germany/ 2021	Retrospective	55	To investigate the ability of PSMA PET to detect metastatic lesions in early CRPC.	^68^Ga-PSMA-11	PSMA PET resulted in positive results in 75% of patients, of whom 45% had M1 status.

nmCRPC: non-metastatic castration-resistant prostate cancer, M1: extra-pelvic metastases; PSMA: prostate-specific membrane antigen; FDG: [^18^F]-fluorodexoyglucose.

**Table 2:** Some useful hints helping to discriminate between metastases and UBU.

Malignant Findings	UBU Findings
Sclerotic/blasting change at any follow-up imaging	PSA < 0.1 ng/mL after curative surgical treatment
SUVmax increase (e.g., ≥30%) on follow-up ^18^F-PSMA-1007 PET, independent from any treatments	Unchanged uptake on follow-up ^18^F-PSMA-1007 PET with or without therapy after >6 months
Treatment-related changes (e.g., reduction in size or increased sclerosis) on follow-up	No longer present on follow-up ^18^F-PSMA-1007
Appearance of metastatic lesion on different imaging techniques (e.g., ^68^Ga-PSMA-11 PET, MRI, bone scan, CT)	Benign aspect on a different imaging modality
Association with typical symptoms of malignancy	Managed as likely benign after clinical evaluation
Association with blood biomarkers (i.e., PSA and ALP increasing/decreasing)	PSA stable or undetectable PSA

**Table 3:** Main findings of the selected papers on double-tracer PET imaging (PSMA ligands plus [^18^F]FDG) in nmCRPC.

Reference	Location Year	Study Type	N. of Patients	Primary Endpoint	Radiotracers	Comment
Michalski et al. [48]	Germany 2021	Retrospective bicenter	54	Assessment of mismatched lesions in mCRPC patients before RLT.	[^68^Ga]-PSMA-11 [^18^F]FDG	33% of mCRPC patients show mismatched PSMA- [^18^F]FDG+ lesions and these patients present shorter OS.
Seifert et al. [30]	Germany, USA 2022	Retrospective	89	Assessment of mismatched lesions in mCRPC patients before RLT.	[^68^Ga]-PSMA-11 ^[18^F]-PSMA-1007 [^18^F]FDG	18% of patients had mismatched lesions between PSMA and [^18^F]FDG PET, however only 3% of patients had mismatch findings not detected using only PSMA PET.
Chen et al. [42]	China 2022	Retrospective	56	To assess metabolic heterogeneity of mCRPC patients	^[68^Ga]-PSMA-11 [^18^F]FDG	[^68^Ga]-PSMA-11 PET/CT showed higher detection rate than [^18^F]FDG PET/CT (75% vs. 51.8%). However, 23.2% of patients showed at least 1 mismatched PSMA- [^18^F]FDG+ lesion.
Güzel et al. [49]	Turkey 2023	Retrospective	71	To investigate the prognostic role of dual-tracer PET imaging in [^18^F]FDG+ mCRPC treated with chemotherapy	^68^Ga-PSMA-11 [^18^F]FDG	Volumetric parameters and Pro-PET scores obtained from dual-tracer PET/CT imaging predict OS in patients with mCRPC treated with taxane chemotherapy. Dual-tracer imaging should be performed in these patients as 78.9% of visceral metastases were PSMA−/FDG+

mCRPC: metastatic castration-resistant prostate cancer; OS: overall survival; RLT: radioligand therapy.

## Data Availability

No new data were created for this study. Data sharing is not applicable to this article.
